# Co-design and implementation of a mHealth intervention targeting fathers and mothers to improve breastfeeding

**DOI:** 10.1186/s12911-023-02125-3

**Published:** 2023-02-08

**Authors:** Kidane Tadesse Gebremariam, Afework Mulugeta, Danielle Gallegos

**Affiliations:** 1 Institute for Physical Activity and Nutrition (IPAN), School of Exercise and Nutrition Sciences, Deakin University, Geelong, Australia; 2 School of Exercise and Nutrition Sciences, Queensland University of Technology (QUT), Victoria Park Road Kelvin Grove, Brisbane, QLD 4059 Australia; 3 School of Public Health, College of Health Sciences, Mekelle University, Mekelle, Ethiopia; 4 Woolworths Centre for Childhood Nutrition Research, Faculty of Health, Queensland University of Technology (QUT), Graham St, South Brisbane, QLD 4101 Australia

**Keywords:** Co-design, eHealth, mHealth, Parents, Breastfeeding, Low-income

## Abstract

**Background:**

Evidence has shown that SMS text message-based health education is effective in improving exclusive breastfeeding. However, there is limited evidence on the development and design of SMS messaging intervention targeting fathers and mothers.

**Method:**

This is the formative assessment and intervention design for a larger trial targeting both fathers and mothers for breastfeeding support in Tigray, Ethiopia. A total of 42 parents of children less than 2 years of age were involved in the design process that also included nutrition experts. We recruited 128 expectant couples to the intervention (1-month antenatally) who continued for 3 months postnatally.

**Results:**

Sixteen messages were developed specific to feeding in the antenatal and postnatal periods. These messages were revised with parents and experts and pretested with parents. Over 4 months 87% of fathers and mothers received 3 or more SMS text messages. All fathers and 97% of mothers read the weekly SMS text messages. Almost 90% of mothers and fathers indicated their willingness to continue to receive SMS text messages related to infant feeding.

**Conclusion:**

Development of SMS based breastfeeding interventions should involve the target population in content design. The role of experts and target population in the co-design process is also crucial.

## Background

Breastmilk is considered the ideal source of nutrition for infants and foundation for health growth and development. For optimal feeding, the World Health Organization (WHO) recommends breastfeeding initiation within the first hour after birth and exclusive breastfeeding for 6 months, with continued breastfeeding to 2 years [1, 2]. Breastfeeding has many benefits for children from infancy into childhood with possible extension into adulthood. There is a dose response; the longer a child is breastfed, the lower the risk of morbidity and mortality, the fewer dental malocclusions, and intelligence is improved as compared to children who are breastfed for shorter periods of time [3]. While exclusive breastfeeding for 6 months is known to be optimal, only 36% of infants globally are fed in this way [4].

There are a range of modifiable determinants which impact maternal breastfeeding practices. These are primarily psychosocial and include; maternal intention to breastfeed, attitudes, self-efficacy to perform breastfeeding, and social support during lactation [5]. These psychosocial factors can be possibly modified through breastfeeding education and promotion interventions [5, 6]. Health education interventions through mobile phones have been shown to improve maternal and child health [7]. Thus, with the growth of mobile phone technologies in low- and middle-income countries (LMIC), mHealth could be a promising strategy to provide breastfeeding promotion and education.

There is growing evidence showing the acceptability of mHealth interventions promoting breastfeeding [8, 9]. However, few interventions have been conducted on their effectiveness for improving breastfeeding practice and duration, very few within a low-income country context and rarely do they include fathers [10]. There is potential for mHealth applications in these low resource areas, given that mobile communication technology is increasing rapidly, with increasing numbers of new mobile subscribers in LMICs [11, 12]. According to the Ericsson Mobility Report, 2021, mobile subscription is expected to grow in in Sub-Saharan Africa, with more than 20% of the global new subscriptions emerging from this region in 2021 [13]. Using mobile coverage and network expansion in Ethiopia as an opportunity, mobile phone technologies can also be considered a mechanism to bring health services, education, and data exchange to communities, living in rural and urban areas [14]. Due to the reliance on 2G/3G technology, higher rates of SMS text messaging are used in LMICs. With minimal costs and higher rates of delivery, SMS text messaging is potentially suitable for interventions in low income countries [15, 16]. It is therefore important to explore innovative mHealth interventions in LMICs like Ethiopia where there are shortages of health personnel, and where there are higher child mortality rates, to create accessible, easy to use, and low-cost health services. The aim of this study was to describe the process of development and evaluation of a mHealth intervention targeting fathers and mothers in a low-income-country.

## Methods

### Setting

The intervention was developed as part of a study exploring the use of a mHealth intervention targeting both mothers and fathers to improve exclusive breastfeeding to 3 months. It took place in Mekelle, the capital city of the regional state of Tigray in February 2018 to March 2019. This was a three-phase process, phase-1: formative assessment; phase-2: co-design of SMS message and pretesting; phase-3: acceptability testing. The detail of the design is described below.

### Phase-1: formative development

The formative assessment for the intervention included exploring the attitudes, knowledge, beliefs, barriers, and enablers of exclusive breastfeeding as well as experiences of mobile phone use, perceived benefits of using a mobile phone, as well as timing, and frequency preferences for receiving SMS text messages for mothers and fathers. Focus group discussions (FGDs) were undertaken with mothers (two groups), and fathers (two groups), resulting in a total of 42 participants. All mothers and fathers had a child less than 2 years of age, access to a personal mobile phone, and were able to read and understand the local language (Tigrigna) and who had provided informed written consent were eligible to participate. Either the mother or the father was included in the FGD but not both. Details of methods and data analysis are described in the formative studies [17, 18].

### Phase-2: co-design of SMS text messages and pretesting

Codesign is a process that involves all stakeholders throughout the design process [19]. This is an active collaboration between researchers, users, and developers to understanding the problem, behaviour, and concept development. Involvement of stakeholders during the design of an intervention helps in developing socially, culturally, and contextually acceptable approaches that work within the contexts of the target populations [20, 21]. Text message content was developed that related to predominantly feeding behaviours relevant antenatally, and in the postpartum period and targeted mothers and fathers separately. The initial text messages were developed based on the findings from the FGD and on best-practice by the authors in English, and then translated to the local language (Tigrigna). A total of 32 messages were developed equally distributed between fathers and mothers with the aim to send a text message once a week for 1 month antenatally and for 3 months after the birth of the baby. These messages were then provided to two paediatricians, two paediatric nurses, and two nutritionists who spoke, read and understood Tigrigna. Experts were asked to provide feedback on the content and wording of the text messages. After incorporating the expert feedback, the SMS text messages were reviewed by an additional five fathers and five mothers who had a child less than 2 years of age, access to a personal mobile phone, and were able to read and understand Tigrigna were recruited from the same two health centres as phase-1. Parents provided their consent to participate in the review discussion. Parents were given a chance to read the contents of the SMS before the discussion and were able to describe how the messages made them feel, the clarity of the message, language used or wording, length of the message, and to provide any additional feedback.

After development of the SMS text messages, the messages were uploaded into a freely available bulk SMS software, FrontlineSMS (http://www.frontlinesms.com). This system was used to send the messages using the EthioTelecom (Ethiopian Telecommunication) network, the sole-provider of telecommunications in Ethiopia. This software was installed on a personal computer and was connected to a mobile phone with a local SIM card, which helped to access the local network. The use of the FrontlineSMS was to enable the sending of bulk SMS. FrontlineSMS text messaging had been used in a previous breastfeeding intervention [22]. The software is also able to track sent, failed, and the pending status of text messages, which is important in managing the process and documenting SMS delivery. This data was collected each time a message was sent. A list of study participant contacts was created with separate groups for mothers and fathers. Messages were sent every Saturday morning to each group in each study arm.

The SMS text messages were pretested with five couples who had a child less than 6 months, could read Tigrinya, and had any type of mobile phone were eligible to participate. Mothers were contacted through their healthcare providers at the health facility during their postnatal clinic visit, immunization, or maternal childcare service appointment. Partners of mothers who agreed to participate were contacted through urban health extension workers. All participants provided informed consent. The participants received a weekly SMS text messages for a period of 1 month. After completing the pre-test, the study participants were requested to provide feedback on simplicity, local and cultural acceptability in terms of language usage and size, and delivery rate of SMS text messaging. Each participant was telephoned to ascertain their satisfaction with the text messaging and if any changes needed to be made.

### Phase-3: intervention

As described in our previous study [23] pregnant mothers and their partners were recruited from three health centres. Participant couples were in their third trimester stage of pregnancy, had access to a personal mobile phone, and were able to read and understand Tigrigna. Each health centre was assigned to one arm of the study; arm 1—mother and father intervention group (both received individual breastfeeding SMS on their own mobile), arm 2—mother only intervention (only the mother received breastfeeding SMS), and arm 3—control group. A total of 128 parents were enrolled in the intervention during their last trimester (43 couples in arm-1, 43 couples in arm-2, and 42 couples in arm-3). Participants in the intervention groups, fathers and mothers in arm 1 and only mothers in arm 2, received 1 month of antenatal SMS, and 3-months of postnatal SMS. Due to adverse outcomes, we lost two couples from each arm. Using face-to-face or telephone interview, 41 fathers from arm—1 and 82 mothers from arms—1 and 2 provided feedback the SMS messages. At the end of each month after giving birth, feedback was obtained on how many SMS messages they received, if they read the message, if they shared the message content with their partner or other people. In addition, at the third month participants also provided feedback on the convenience of the SMS based breastfeeding education, willingness to continue to receive breastfeeding SMS text messages and how influential the SMS based breastfeeding intervention was about breastfeeding.

### Data analysis

Data were entered IBM SPSS Statistics version 23 (IBM Corp. Released 2015. IBM SPSS Statistics for Windows, Version 23.0. Armonk, NY: IBM Corp). Descriptive statistics were conducted to describe message delivery and satisfaction for fathers and mothers.

## Results

### Phase-1: content of the intervention

The content of the SMS breastfeeding education was developed after conducting an explorative qualitative study with fathers and mothers who had a child less than 2 years of age. Sixteen weekly messages for fathers and 16 messages for mothers were developed that were tailored to the antenatal and postnatal periods (Table 1).

**Table 1:** Antenatal and postnatal SMS text messages sent to fathers and mothers

Time	Fathers	Mothers
Antenatal SMS messages	Have you talked to your wife about breastfeeding your baby?	It may take a day or two for your milk to come in don’t give anything else but colostrum
	Breastmilk only will make your baby grow big, strong and smart	Always give your baby access to your breast so they can feed when they are hungry or thirsty
	The first milk colostrum is good for baby it will help the baby fight infection	The first milk colostrum is good for baby it will help the baby fight infection
	Ask the health workers to put your baby to your wife’s breast within an hour of giving birth	Let the baby suckle at your breast to increase milk supply
*Postnatal SMS messages*		
Month one	Breast milk has everything, and it is clean so that it helps brain development, to build the body, for health and its good for everything	When the baby has stomach, pain bring the baby to the health facility, don’t give fenugreek
	Encourage your wife to breastfeed whenever the baby is hungry	Breastmilk is clean and safe and has enough water
	All your baby needs for the first 6 months is breastmilk, don’t give other food or liquid	Give your baby all of the breastmilk in one breast before starting on the other breast
	Feeding only breastmilk is important for your baby to grow big and strong	Ask your partner to bring you food and provide support
Month two	No water just breastmilk—water may give your baby diseases	Breastmilk has everything, and it is clean so that it helps brain development, to build the body, for health and its good for everything
	Help your wife, bring her a drink of water, soup or milk while she is breastfeeding	Breastmilk will protect your baby from diarrhea
	Exclusive breastfeeding can protect from breast and cervical cancer	Exclusive breastfeeding can protect from breast and cervical cancer
	Tell the housemaid/grandmothers no water or food just breastmilk	All your baby needs for the first 6 months is breastmilk, don’t give other food or liquid
Month three	Help your wife express breastmilk into a cup if she is going out	Even if the baby is smelling don’t give food, wait until they are 6 months old
	Help your wife to breastfeed by doing the shopping	Don’t stop breastfeeding, you can overcome all challenges
	Encourage your wife to let the baby suckle at her breast to increase milk supply	Express breastmilk into a cup if you are going out
	Make sure your wife eats enough food—serve your wife if she is breastfeeding	Providing water, foods and liquids other than breastmilk will expose the baby to disease

### Phase-2: review of SMS text messages

After reviewing the SMS text messages the health care providers provided constructive comments in terms of wording which were incorporated into the messages. The comments related to the use of Tigrigna words which are not commonly used in the study area, and the use of Amharic language and changes were subsequently made. In addition, recommendations about including a reminder about immunizations was also raised but not included in the intervention due to the focus on feeding. The health professionals strongly agreed with the content, cultural suitability, and appropriateness in terms of antenatal and postnatal periods. Consequent to the review from experts, the revised messages were reviewed by five mothers and five fathers who had a child less than 2 years of age. Like the experts, parents commented on the use of language and wording, and these were addressed to enhance readability. Parents agreed with the content covered, clarity of messages and the ease of understanding. Participants indicated that all messages arrived, that the timing of messages was suitable. Based on the feedback from the FGD all messages were sent on a Saturday [18], they were easy to read and understand. As a result no additional modifications were required.

### SMS text message delivery and satisfaction

**Fig. 1:**
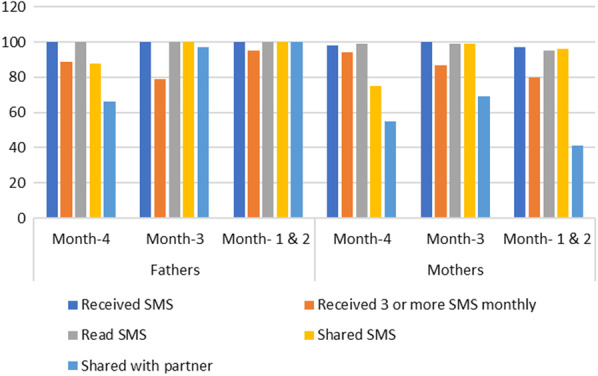
Description of SMS text messages delivery by month

**Fig. 2:**
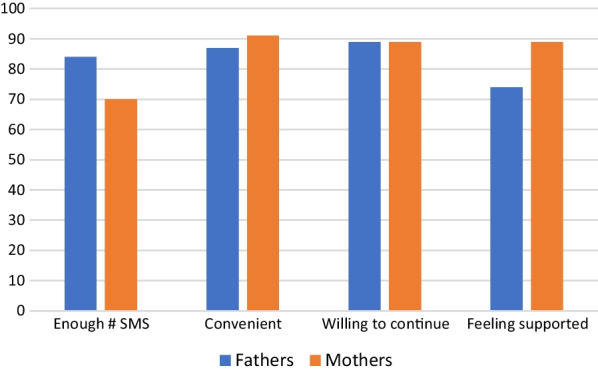
Participants satisfaction on the SMS text message intervention

Over the 4 months of the intervention 87% of fathers and mothers received 12 or more SMS text messages. All fathers, and 97% of mothers read the weekly SMS text messages. A majority (80%) of fathers, and 55% of mothers shared the SMS text messages with their partners’ (Fig. 1). In addition, 89% of mothers and fathers indicated their willingness to continue to receive SMS text messages related to infant feeding (Fig. 2).

## Discussion

This paper discusses the development and implementation of a SMS based breastfeeding education intervention for fathers and mothers in a low resource setting. This included, co-designing the content of the health education intervention, pretesting the intervention and delivery mode, and gathering data on the successful delivery of SMS text messages, and satisfaction over 4 months.

Involving stakeholders during design of SMS text message content is useful to ensure a range of views of the target population are included to ensure the context of the intervention is taken into consideration before implementation [20]. In this instant, formative studies were conducted to explore the major areas of to target for breastfeeding promotion [17] and the possibility for using SMS text messages for breastfeeding education [18] in the target population. Involvement of stakeholders during the intervention design assists in developing socially, culturally and contextually acceptable approaches [21]. The major steps for developing a text messaging program include conducting formative research using FGD for insights into the target audience and health behaviour, designing the text messages, pretesting the text messaging program concept and messages, and revising the text messaging program [24].

In the current study, co-design was the heart of the content development process. After the formative assessments [17, 18] the SMS text messages were revised by nutrition experts on the content, language and appropriateness for the target population. Subsequently, content of the SMS text messages were also revised by fathers and mothers to confirm their appropriateness in terms of cultural suitability, language use and suitability for mobile phone. Pretesting is a method of maintaining data quality and early identification of potential problems prior to the intervention [24]. In the current study, content of the intervention was pretested with fathers and mothers to understand ease of readability, SMS message size suitability and delivery. In addition, the pretesting phase was crucial to test the FrontlineSMS platform to manage sent and pending messages. Incorporating the feedback received from participants in the pretesting is important to improve the messaging and features of the program [25]. Therefore, involving the target population in the pretesting phase and in the design and development of SMS text message interventions is important so as to alleviate potential problems prior to intervention implementation.

One of our formative assessments indicated the increased acceptability of SMS text messages for breastfeeding education if they were from experts [18]. As the SMS messages were linked to the health centre couples were attending, we found higher satisfaction levels and a high willingness to continue to receive SMS text messages. In addition, participants indicated that the SMS text messages were convenient, and they felt supported when they received SMS text messaged related to breastfeeding. The number of SMS text messages received relates to the dose of the intervention and there is evidence this could effect engagement with the intervention [26]. Our formative assessment showed that a weekly SMS text message was enough so that parents were engaged in but not irritated by the intervention [18], as a result the SMS text messages received high reading rates of SMS text message and higher agreement on the amount of SMS text messages received. The success of the SMS text messages and their impact on improving exclusive breastfeeding rates in the first 3 months was evident in the significant increases in exclusive breastfeeding in both the mother and fathers arm (85%, *p* = 0.01), and mothers-only arm (80%, *p* = 0.04), compared to the control group where only 60% of participants achieved exclusive breastfeeding [27].

The study has significant strengths including that it is one of only a few that involve fathers and mothers. In addition, all the important components of design and implementation were incorporated, including the involvement of the target population in the content development, co-design, pretesting, and implementation. However, there are limitations. A cost–benefit analysis was not undertaken and so costs associated with the SMS intervention were not analysed. In addition, this study was conducted in a state capital city, so that the findings may not be able to be generalized to rural areas.

## Conclusion

In conclusion, the development of SMS based breastfeeding interventions should involve the target population (health professionals and targeted recipients) in content design and in the co-design process to improve intervention efficacy. In addition, pretesting and incorporating feedback improves the acceptability and readability of the SMS text messages. Involving all those steps improved the acceptability and engagement of participants with the intervention. Co-designing interventions and paying attention to factors that could influence intervention acceptability and suitability ensures interventions are fit-for-purpose and improves their effectiveness in changing behaviours that influence health, in this case breastfeeding.

## Data Availability

The datasets generated and/or analysed during the current study are not available publicly but are available on reasonable request from the corresponding author.

## References

[1] World Health Organization (2001). Global strategy for Infant and Young Child Feeding, in The optimal duration of exclusive breastfeeding.

[2] World Health Organization (2009). Model chapter for textbooks for medical students and allied health professionals, in Infant and young child feeding.

[3] Cesar V (2016). Breastfeeding in the 21st century: epidemiology, mechanisms, and lifelong effect. Lancet.

[4] World Health Organization. Exclusive breastfeeding under 6 months: Global Health Observatory data repository. 2014 [cited 2016 12/09]; Available from: http://apps.who.int/gho/data/view.main.NUT1710?lang=en.

[5] Meedya S, Kathleen F, Ashley KK (2010). Factors that positively influence breastfeeding duration to 6 months: a literature review. Women and Birth.

[6] Dennis C-L (2003). The breastfeeding self-efficacy scale: psychometric assessment of the short form. J Obstet Gynecol Neonatal Nurs.

[7] Lee SH (2016). Effectiveness of mHealth interventions for maternal, newborn and child health in low–and middle–income countries: systematic review and meta–analysis. J Glob Health.

[8] Myat PH (2016). A formative study to inform mHealth based randomized controlled trial intervention to promote exclusive breastfeeding practices in Myanmar: incorporating qualitative study findings. BMC Med Inf Decis Mak.

[9] Huang M (2007). Evaluating effects of a prenatal web-based breastfeeding education programme in Taiwan. J Clin Nurs.

[10] Tadesse K (2018). Effectiveness of breastfeeding interventions delivered to fathers in low- and middle-income countries: a systematic review. Matern Child Nutr.

[11] Union IT. Mobile overtakes fixed: Implications for policy and regulation. 2003: Canada.

[12] Orbicom-ITU, From the digital divide to digital opportunities: measuring infostates for development. 2005: Canada.

[13] Ericsson, Ericsson Mobility Report: on the pulse of the networked society, P. Cerwall, Editor. JUNE 2015: Sweden.

[14] Ethiopian Federal Ministry of Health, Information Revolution Roadmap. 2016: Addis Ababa. p. 12–13.

[15] Bastawrous A, Hennig B, Livingstone I (2013). mHealth possibilities in a changing world: distribution of global cell phone subscriptions. JMTM.

[16] Beratarrechea A (2014). The impact of mobile health interventions on chronic disease outcomes in developing countries: a systematic review. Telemed E-Health.

[17] Gebremariam KT (2020). Exploring the challenges and opportunities towards optimal breastfeeding in Ethiopia: a formative qualitative study. Int Breastfeed J.

[18] Gebremariam KT (2020). Could mobile phone text messages be used for infant feeding education in Ethiopia? A formative qualitative study. Health Informatics J.

[19] Sanders EBN, Stappers PJ (2008). Co-creation and the new landscapes of design. CoDesign.

[20] Croot L (2019). Developing interventions to improve health: a systematic mapping review of international practice between 2015 and 2016. Pilot Feasibility Stud.

[21] Vaughn LM (2016). See what we say: using concept mapping to visualize Latino immigrant’s strategies for health interventions. Int J Public Health.

[22] Hong J (2014). Effect of shortmessage service on infant feeding practice findings from a community-based study in Shanghai, China. JAMA Pediatr.

[23] Gebremariam KT (2021). A cross-sectional comparison of breastfeeding knowledge, attitudes, and perceived partners’ support among expectant couples in Mekelle, Ethiopia. Int Breastfeed J.

[24] Abroms L (2015). Developing and pretesting a text messaging program for health behavior change: recommended steps. JMIR Mhealth Uhealth.

[25] Ybarra ML (2014). Process evaluation of a mHealth program: lessons learned from Stop My Smoking USA, a text messaging-based smoking cessation program for young adults. Patient Educ Couns.

[26] Szinay D (2020). Influences on the uptake of and engagement with health and well-being smartphone apps: systematic review. J Med Internet Res.

[27] Gebremariam KT, Mulugeta A, Gallegos D (2023). Theory-based mHealth targeting fathers and mothers to improve exclusive breastfeeding: a quasi-experimental study. Int Breastfeed J.

